# GNOSIS: an R Shiny app supporting cancer genomics survival analysis with cBioPortal

**DOI:** 10.12688/hrbopenres.13476.1

**Published:** 2022-01-18

**Authors:** Lydia King, Andrew Flaus, Simone Coughlan, Emma Holian, Aaron Golden

**Affiliations:** 1 SFI Centre for Genomics Data Science, National University of Ireland, Galway, H91 TK33, Ireland; 2 School of Mathematical & Statistical Sciences, National University of Ireland, Galway, H91 TK33, Ireland; 3 Centre for Chromosome Biology, School of Natural Sciences, National University of Ireland, Galway, H91 TK33, Ireland

**Keywords:** Cancer Genomics, cBioPortal, Precision Oncology, Statistical Analysis, Survival Analysis, Data Exploration, R, RShiny app

## Abstract

Exploratory analysis of cancer consortia data curated by the cBioPortal repository typically requires advanced programming skills and expertise to identify novel genomic prognostic markers that have the potential for both diagnostic and therapeutic exploitation. We developed GNOSIS (GeNomics explOrer using StatistIcal and Survival analysis in R), an R Shiny App incorporating a range of R packages enabling users to efficiently explore and visualise such clinical and genomic data. GNOSIS provides an intuitive graphical user interface and multiple tab panels supporting a range of functionalities, including data upload and initial exploration, data recoding and subsetting, data visualisations, statistical analysis, mutation analysis and, in particular, survival analysis to identify prognostic markers. GNOSIS also facilitates reproducible research by providing downloadable input logs and R scripts from each session, and so offers an excellent means of supporting clinician-researchers in developing their statistical computing skills.

## Introduction

Cancer diagnosis, classification and treatment generally follows an integrative approach combining clinical features and tissue-based biomarkers ^1, 2^. In recent years, there has been an increased interest in using genetic testing to guide treatment decisions, predict patient response and determine likely prognoses for cancers associated with specific pathogenic variants ^3^. Such a precision oncology paradigm has been fostered by the extensive efforts of many cancer genomics consortia, yielding extraordinarily rich repositories of genomic and associated clinical data of hundreds to, in some cases, thousands of cancer patients ^4, 5^.

Summary clinical and cancer genomic data are available from a number of consortia websites, with cBio Cancer Genomics Portal ( cBioPortal) ^6, 7^ offering one of the best known and regularly accessed consolidated curations for multiple consortia; cBioPortal provides both graphical user interface (GUI)-based and representational state transfer (RESTful) mediated means for researchers to explore clinical and genomics data. However, cBioPortal’s exploratory capabilities have their limitations, requiring the implementation of a more sophisticated ‘off site’ analysis that typically requires significant prior programming experience. This remains arguably the greatest barrier for many clinician-researchers wishing to explore hypotheses in precision oncology.

An ideal solution to this bottleneck would be the availability of a software environment supporting the integration of cBioPortal-hosted data products, their visualisation and tractable manipulation using standard biostatistical methodologies. Such an environment would provide a convenient means of testing exploratory hypotheses, particularly those assessed in the context of survival analysis, in a way that would be both reproducible and interpretable. Based on our experience as part of a recent study ^8^ to investigate whether survival outcomes are associated with genomic instability in luminal breast cancers, we developed a software infrastructure using R to facilitate such exploratory work. Working with the Molecular Taxonomy of Breast Cancer International Consortium ( METABRIC) ^9^ summary clinical and genomic copy number alteration (CNA) data obtained from cBio-Portal, we tailored this codebase to support GUI interactivity, and deployed it as an R Shiny app called GNOSIS (GeNomics explOrer using StatistIcal and Survival analysis in R).

GNOSIS leverages a number of R packages. The GUI front end employs ‘tabs’ for data upload, initial exploration, data subsetting and recoding, a range of visualisations, comprehensive survival analysis, association testing and mutation analysis. Furthermore, GNOSIS has a user-driven point-and-click interface that logs all user activity to facilitate reproducibility, and ultimately enables the statistical analysis and incorporation of multiple and diverse genomic features with patient data in a research or clinical setting.

GNOSIS provides a tractable means for clinician-researchers with a background in biostatistics to effectively engage with complex cancer genomics data, to experiment with exploratory hypotheses in a more intuitive way that would require greater expertise working at the command line, and to have a record of all activities from which subsequent, more focused and nuanced analyses can be based. GNOSIS also offers great potential in supporting the teaching of biostatistical methodologies relevant to clinical genomics applications. Given its open source basis and foundation in the R statistical programming environment, GNOSIS also offers a means for third parties to enhance and develop its functionality for broader clinical genomics.

## Methods

### Implementation

***Overview.*** GNOSIS was initially developed to enable the exploration, visualisation and analysis of the METABRIC clinical and CNA summary data obtained from cBioPortal, as detailed in King *et al*. (2021) ^8^, and the following description of its operational capabilities have their basis in that study. Although GNOSIS accepts multiple file types, including comma-, semicolon- or tab-delimited, the default settings are suited to files downloaded from cBioPortal. If users wish to upload clinical or summary genomics data files from other sources, care should be taken to set appropriate default values. GNOSIS leverages a number of R packages, primarily shiny, tidyverse, ggplot2, survival, survminer, rpart, partykit and maftools ^10– 18^. A full list is provided in the Operation subsection. It allows users to carry out a comprehensive visual exploration and statistically robust survival analysis in a fast, simple and reproducible way, and we illustrate GNOSIS functionalities by referring to example screenshots of its operation at various relevant steps of the analysis documented in King *et al.* (2021) ^8^.

**Figure 1.**
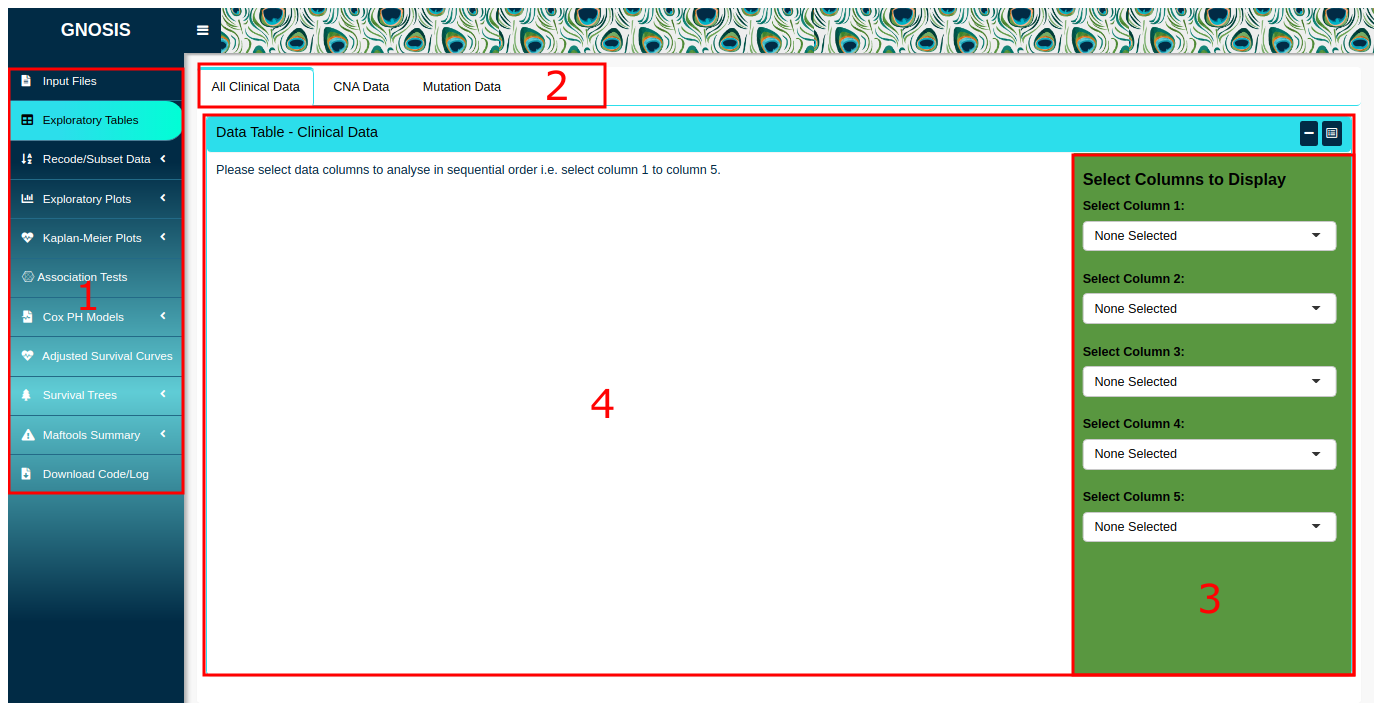
GNOSIS GUI with highlighted interface elements. (1) The Exploratory Tables tab is selected in the tab sidebar. (2) Within tab panels allowing multiple operations to be carried out and viewed in the one tab. (3) Box sidebar allowing users to select inputs, alter arguments and customise and export visualisations. (4) Viewing panel displaying output.

***Data upload and formatting.*** In Figure 1 we show the GNOSIS front-end with the specific entry points and ‘tabs’ highlighted. GNOSIS accesses files locally on the user’s file system, and in its default configuration, is optimised to use data files downloaded from cBioPortal. In the Input Files tab, users are provided with a space to upload the clinical patient and sample data, summary CNA data and mutation data. Whilst we have configured GNOSIS to work exclusively with both CNA and mutational data files, modification of the codebase allows users to reconfigure the GNOSIS GUI to import other genomic tracks from cBioPortal, as required. A preview of the data is provided in the GNOSIS viewing panel to ensure that the data has been read in correctly. It should be noted that the clinical patient and sample data should contain a column named "PATIENT_ID" and the CNA data should contain a column called "Hugo_Symbol". As these are core named data types for all subsequent analytics, warnings will be produced and downstream analysis will not be possible if they are missing.

Once the data is uploaded, further exploration of specific columns can be done using the Exploratory Tables tab, where up to five columns can be selected in the box sidebar and viewed. The columns should be selected in sequential order; if this is not adhered to an error will be displayed.

Before more extensive data exploration and analysis, users are encouraged to carry out pre-processing to ensure data is in the desired format using the Recode/Subset Data tab. Users are provided with a workspace to view the variables present in the data, their type and factor levels. Users can change variables to numeric or factors using the box sidebar, which contains a space to select relevant variables.

Subsequently, users can subset the data based on up to three categorical variables and carry out survival variable recoding. In cases where CNA data is uploaded, users may produce and segment CNA metrics for each patient, as well as select and extract specific genes for further analysis. After each operation, the space to explore variable information is updated. This allows users to confirm their alterations have been implemented correctly. These operations ensure that the data is in the correct format for downstream analysis.

**Figure 2.**
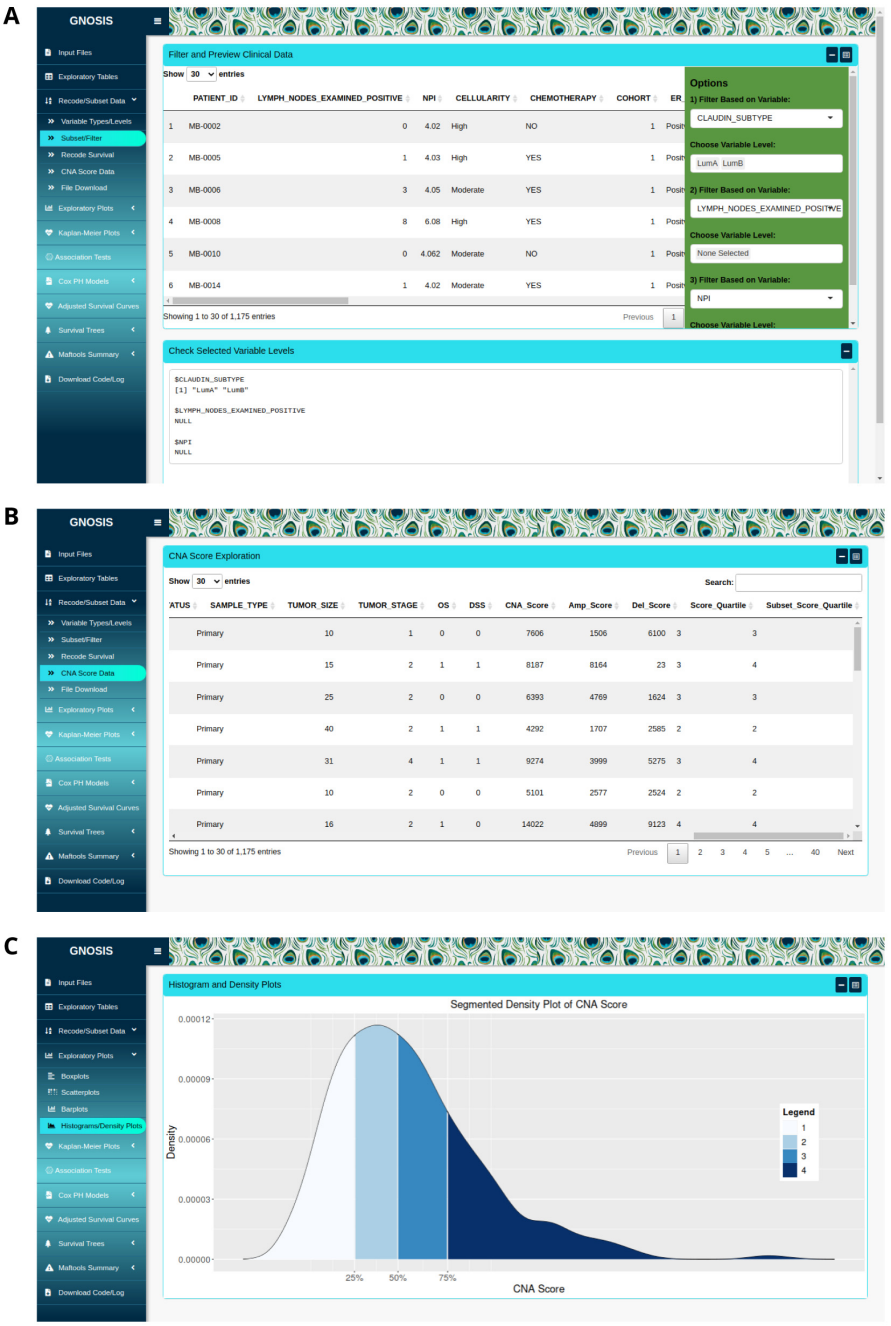
( **A**) The Recode/Subset tab, where data is being subsetted based on subtype, luminal A and luminal B subtypes selected. ( **B**) The dataset after CNA metrics have been calculated and quartile segmented. ( **C**) A density plot of the resulting quartile segmentation.

To allow users to save their formatted file, a space within the tab is provided. If this exported data is uploaded to GNOSIS, formatting of categorical data may have to be carried out again due to the default stringsAsFactors argument implemented when uploading data in R. In Figure 2 (A), we show how a given data file can be examined and filters applied to extract a subset, and in Figure 2 (B), the resulting subset following calculation of CNA metrics, including absolute CNA score, amplification score and deletion score for each patient, and subsequent quartile segmentation of the CNA scores is shown.

***Data visualisation.*** Within the Exploratory Plots tab, GNOSIS provides users with a range of visualisations including boxplots, scatterplots, barplots, histograms and density plots. For example, in Figure 2 (C), a set of patient genome absolute CNA scores can be segmented - and labelled as such - into multiple equally-sized groups. These visualisations are implemented using the R package ggplot2 ^12^. This way, clinical and genomic data can be interrogated and visualised separately or in combination. For each visualisation, users can use the box sidebar to select which columns to interrogate, choose whether to include NA values in the plots, choose whether to display a legend and change the plot title, x- and y-axis titles and legend titles, among others. Further options available to users include the ability to produce boxplots where the sample size is reflected in the width of the boxplot, produce scatterplots coloured by an additional variable, and to produce plain, segmented and faceted histograms and density plots. Users can also download all the resulting plots as .pngs in specified dimensions.

***Statistical and survival analysis.*** The primary function offered by GNOSIS is statistically robust survival analysis. GNOSIS contains several step-wise tabs to provide a complete survival analysis of the data under investigation.

**Figure 3.**
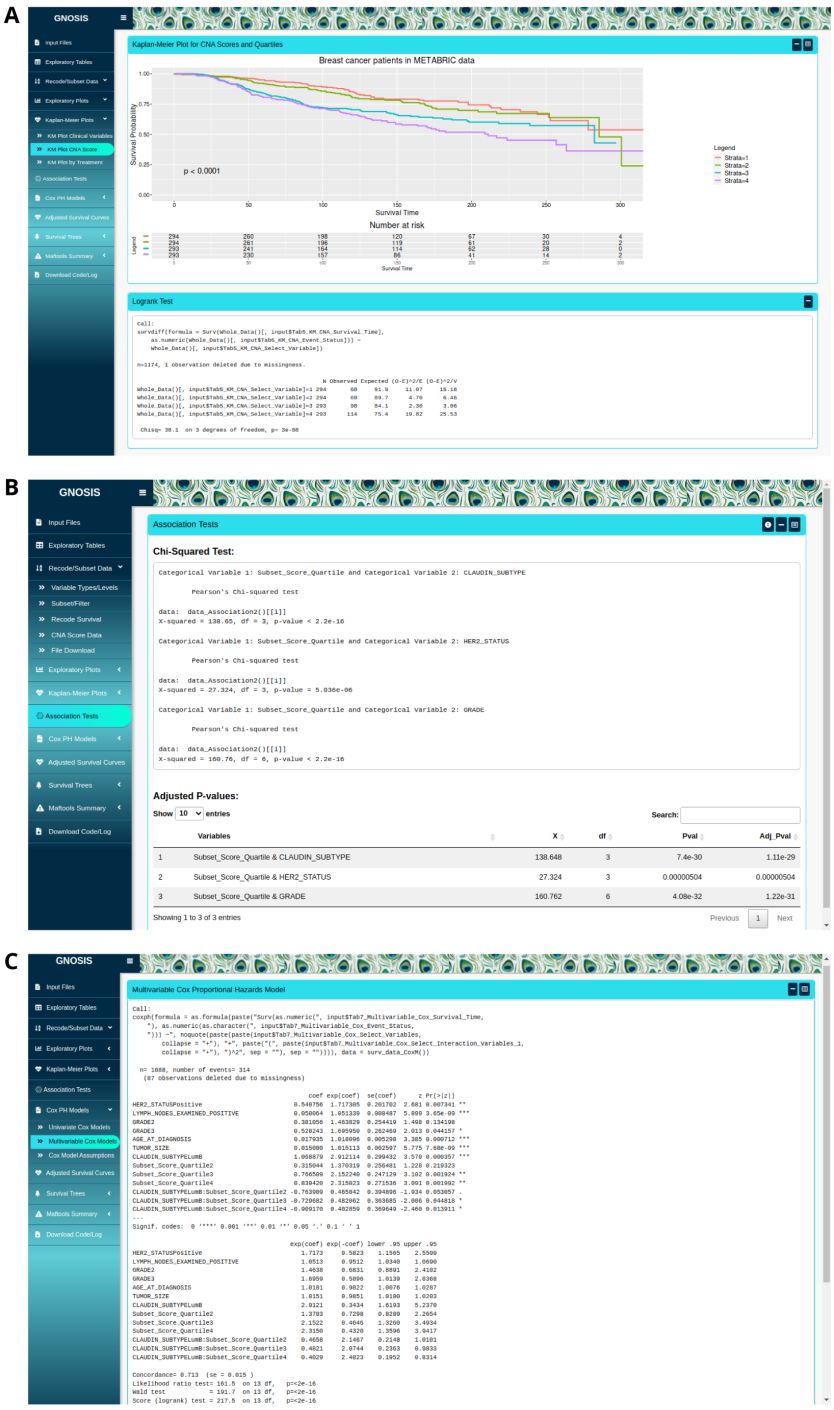
( **A**) Kaplan-Meier (KM) plot for luminal breast cancer disease specific survival (DSS) for each CNA quartile group. The p-value associated with the logrank test and a risk table displaying the number of patients at risk at each time interval is displayed. ( **B**) Example of a *χ* ^2 ^ analysis of the data, individual *χ* ^2^ tests displayed in top box and table with adjusted p-values displayed in bottom box. ( **C**) Example of an implementation of a multivariable Cox model.

Initially the Kaplan-Meier (KM) Plots tab provides survival plots and the corresponding logrank tests to identify survival-associated categorical variables, both visually and statistically Figure 3 (A). The Kaplan-Meier Plots tab contains three sub-tabs which provide users with spaces to produce KM plots and logrank tests for selected clinical variables, for segmented CNA variables and for variables of interest split on treatment assignment (i.e. Where patients received different treatments, e.g. split into patients who received radiotherapy and patients who did not). Within each sub-tab, the interface allows users to indicate which columns contain the survival time, event status (Overall Survival (OS) or Disease Specific Survival (DSS)) and the variable of interest. It should be noted that when producing KM curves for variables split by treatment assignment the selected treatment variable must be coded as a binary YES/NO. These KM curves can be customised and exported as .pngs using the sidebar options.

The Association Tests tab uses association tests to identify variables that are linked to each other and enables users to identify potential confounding variables in the analysis. Variable selection is done within the box sidebar and statistical association tests available include the *χ* ^2^ test, Fisher’s exact test, simulated Fisher’s exact test, ANOVA, Kruskal-Wallis test, pairwise t-test and Dunn’s test. The *χ* ^2^ test is used to assess the association between two categorical variables with sufficient cell sizes in the two-way table of categorical variables ( Figure 3 (B)). Fisher’s exact test can be used in the case where any cell size is sufficiently small. ANOVA can be used to test whether there is a difference in means between groups and the Kruskal-Wallis test may be used in the situation where the assumptions of the ANOVA test are violated. Pairwise comparisons using t-tests and Dunn’s test are also available. In all cases, results of each individual association test are displayed alongside the adjusted p-values calculated using the Benjamini-Hochberg p-value adjustment. It is important that users make sure they run the appropriate statistical tests for the question of interest, that all relevant assumptions are met and that the output is interpreted correctly.

In the Cox Proportional Hazards (PH) models tab, users are provided with a workspace to produce both univariate and multivariable Cox models to identify survival-associated variables, and test the assumptions of these models using graphical diagnostics based on the scaled Schoenfeld residuals ( Figure 3 (C)). The Cox PH model is a regression model commonly used to investigate the association between the survival time of patients and predictor variables. The Cox PH model works for both continuous and categorical variables and extends survival analysis methods to simultaneously assess the effect of several risk factors on survival time. To produce the univariate and multivariable Cox models, the box sidebar enables the selection of the columns that contain the survival time, event status and the variables to be included in the models. The output of each univariate Cox model is displayed along with a summary table containing the adjusted p-values calculated using the Benjamini-Hochberg p-value adjustment. The validity of the PH assumption of each multivariable model fitted can be assessed by producing visualisations based on the scaled Schoenfeld residuals. The Schoenfeld residuals are independent of time, and therefore a plot displaying a non-random pattern against time indicates that the PH assumption may be violated. Where a non-significant relationship between residuals and time is observed, the PH assumption is met. Again, these plots can be customised and exported in portable network graphics format (PNG).

**Figure 4.**
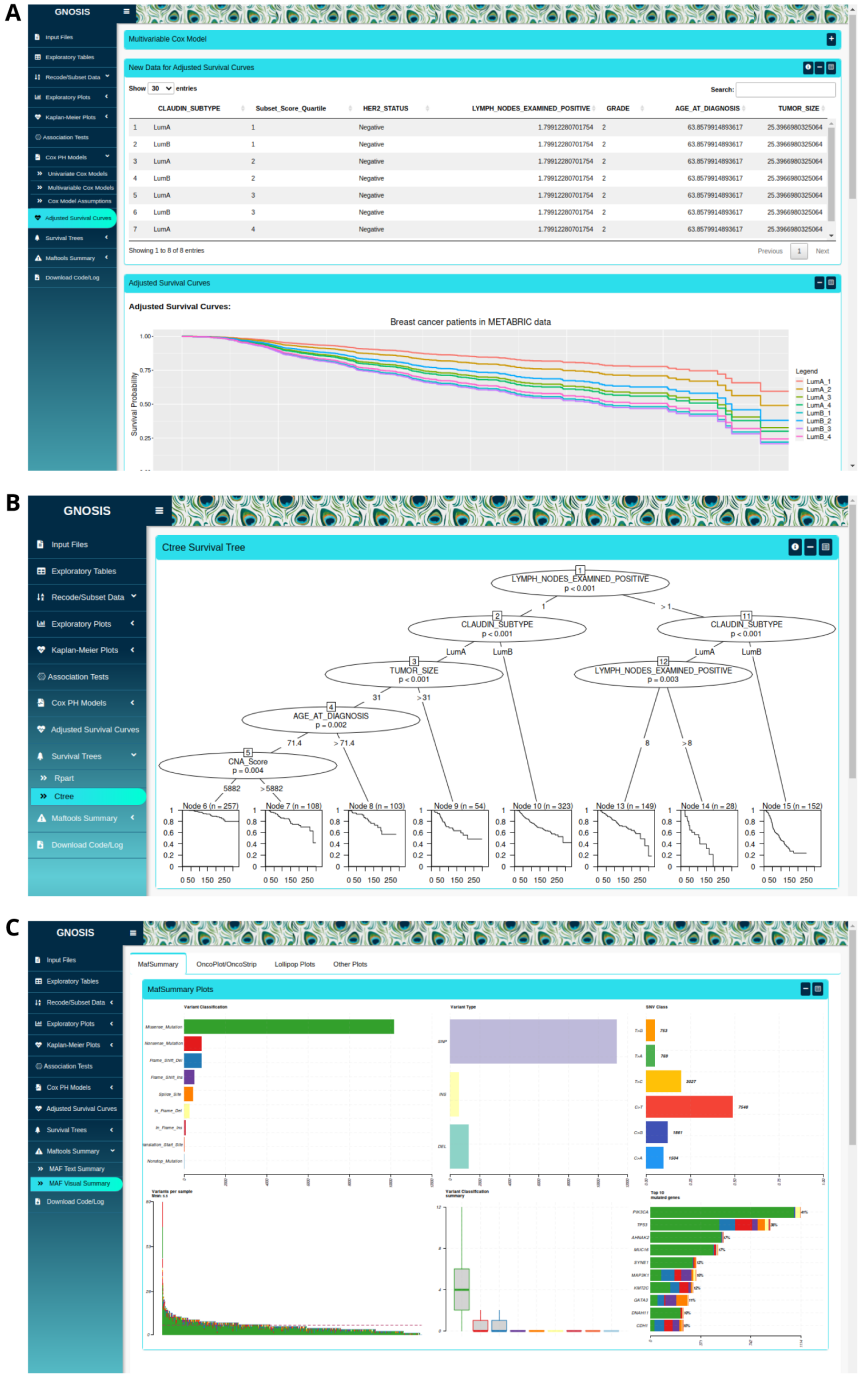
( **A**) Output showing format of new data and adjusted survival curves corresponding to the previous multivariable Cox model. ( **B**) Example output of a ctree survival tree analysis. ( **C**) Sample output from use of the maftools package, a MafSummary plot is displayed.

Following multivariable Cox model selection, users are given the option to produce corresponding adjusted survival curves, which are survival curves adjusted for the covariates in the multivariable Cox model. Within the Adjusted Survival Curves tab, users are provided with a workspace to view the multivariable Cox model that was fitted in the previous tab. This will aid users when creating the new data frame needed to produce the adjusted survival curves. Users are provided with a space to set up the new data frame including the grouping variable, variable of interest and the variables to be kept constant ( Figure 4 (A)). It should be noted that all variables included in the multivariable Cox model should be included in the new data frame. Plots displaying all the adjusted survival curves and adjusted survival curves split based on grouping variable level are displayed for users to view, customise and download in PNG format.

In the case where the PH assumption of the multivariable Cox model is violated, users can apply recursive partitioning survival trees available in the Survival Trees tab. Users can use the rpart or ctree ^15– 17^ algorithms with customised argument parameters to produce survival trees containing one or more variables along with the corresponding KM curves ( Figure 4 (B)). Users are provided with a workspace to select the survival time, event status and variables of interest, and information on each of the customisable arguments is given by pressing the information button located at the top of the box. Similar to previous tabs, the survival trees and accompanying KM curves can be exported with specified plot width and height. It should be noted that the ctree algorithm will only work where the selected categorical variables are in factor form.

***Mutation analysis.*** An additional function of GNOSIS is the ability to summarise, analyse and visualise mutation annotation format (MAF) files using maftools ^18^. MAF files are used to store detected somatic variants and are usually provided as part of the cBioPortal downloads. The Maftools Summary tab in GNOSIS allows users to view the MAF summary, sample summary, gene summary and summary of the associated clinical data, if available. If clinical data are provided users need to make sure the column named "Tumor_Sample_Barcode" is present. These summaries provide a basic view of the uploaded MAF file and contain information on the number of mutations, type of mutations and genes affected by these mutations. The Maftools Summary tab also enables users to examine the mutational landscape of the tumours in a graphical way. The plots available include MAF summary plots which display the number of variants in each sample as a stacked barplot and variant types as a boxplot summarized by variant classification ( Figure 4 (C)). GNOSIS also contains panels for oncoplots, oncostrips, graphs displaying transition and transversion rates, lollipop plots for up to three genes simultaneously, mutation load plots and somatic interaction plots, all derived from the original *maftools* package. Other functions of this tab include allowing users to customise and export these plots in PNG format with specific dimensions.

### Reproducible research

**Figure 5.**
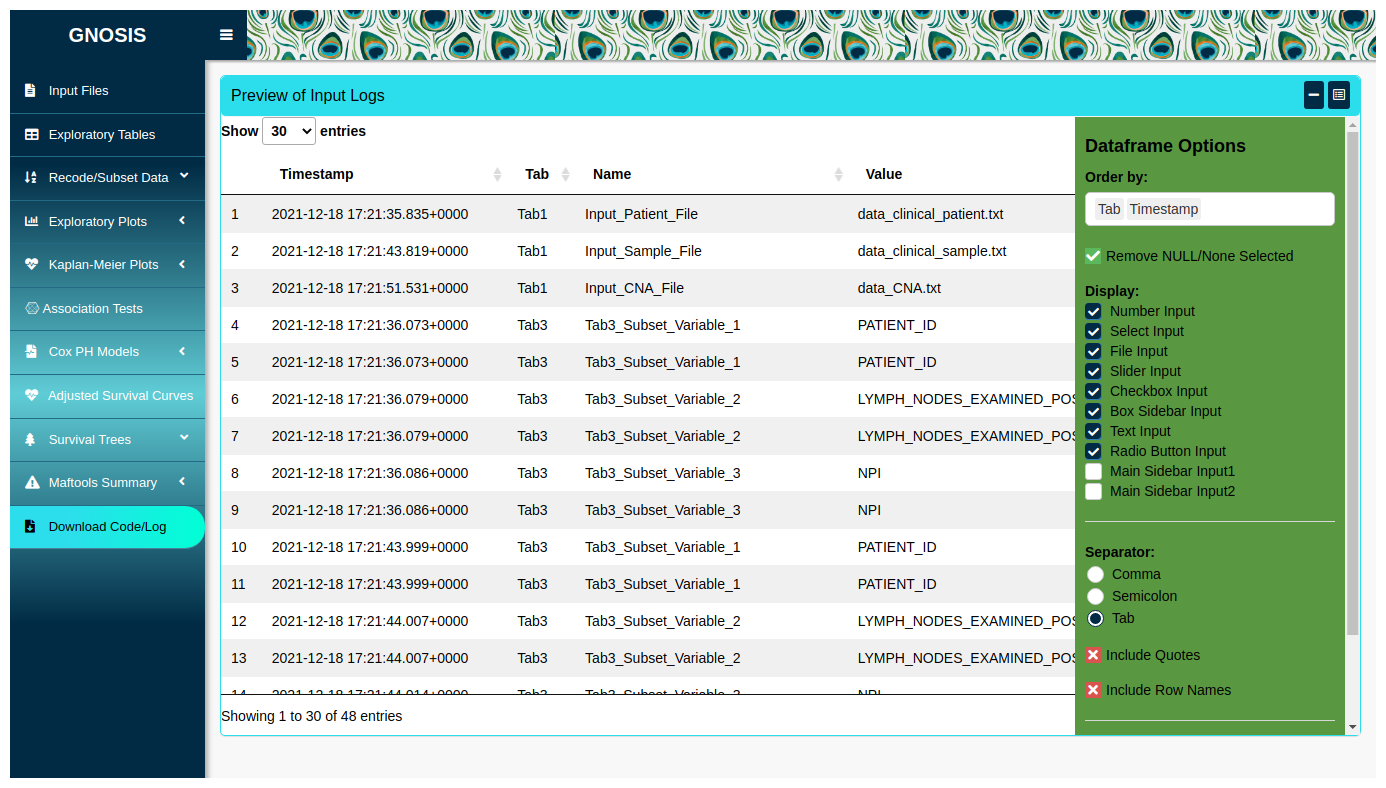
Dataframe containing log of inputs selected, which can be downloaded as a .txt file. Option to download R script containing code run in App also available.

GNOSIS facilitates reproducible research by providing a Download Code/Log tab where users can view and download a log containing information on all the inputs selected throughout the session, as well as downloading an R script containing code to reproduce the outputs displayed in the app ( Figure 5).

### Operation

GNOSIS is available on shinyapps.io and GitHub. This enables users to access GNOSIS via a web browser or run GNOSIS locally by downloading, extracting and launching the app manually in RStudio, or running the app in RStudio using: shiny::runGitHub(repo=‘GNOSIS’,username = ‘Lydia-King’,ref="main"). The latter is recommended due to resource limitations imposed by the operators of the shinyapp.io website.

GNOSIS was originally developed using R version 3.5.1 but due to subsequent package updates, it now works on R versions _≥ _4.0.0. GNOSIS depends on the packages, BiocManager, shiny, shinymeta, shinydashboard, dashboardthemes, shinydashboardPlus, shinyWidgets, shinycssloaders, shinylogs, fontawesome, DT, tidyverse, ggplot2, fabricatr, reshape2, operator.tools, rpart, rpart.plot, partykit, coin, survminer, survival, stats, rstatix, DescTools, car, R.utils, RColorBrewer and maftools ^10– 39^, which are automatically installed and loaded when running GNOSIS manually from RStudio. Should GNOSIS be run using the runGitHub() function, shiny must be installed beforehand.

## Use cases

GNOSIS was originally developed as part of a study to implement an exploratory and statistically robust survival analysis on the METABRIC luminal breast cancer cohort ^8^, and was pivotal in our ability to efficiently determine that CNAs reflecting genomic instability in luminal breast cancers are associated with survival. This work demonstrated both the utility and capability of this analytic ecosystem to facilitate oncogenomic analysis, and motivated us to make it available to the research community, to both use and further enhance, as appropriate.

The data utilised in the study ^8^ is available for download on cBioPortal as well as Zenodo ( *Underlying data ^40^*). Demonstration videos providing a walkthrough of GNOSIS are also provided on Zenodo ( *Extended data ^41^*). An Rmarkdown file and example R script containing the code to run the analysis presented are available on the project’s GitHub. 

We have also provided a subset of the METABRIC data used as part of King *et al*. (2021) ^8^ in the project’s Zenodo repository to facilitate those users interested in exploring the capabilities of GNOSIS using the shinyapps.io app.

## Conclusions

We have developed GNOSIS, an R Shiny app that supports the tractable and efficient exploratory analysis of cBioPortal clinical and genomic data products in a reproducible manner. Our experience with GNOSIS demonstrates its potential in helping researchers and clinicians in the analysis of archived consortia studies curated and accessible from cBioPortal, optimising the identification of variables and scores that have prognostic value and can aid in the identification of patients with a greater risk of lethal disease. Furthermore, GNOSIS’ design and open-source basis makes it amenable to further development and enhancement by interested members of the community.

## Data availability

### Underlying data

Zenodo: Data associated with "Survival outcomes are associated with genomic instability in luminal breast cancers", https://doi.org/10.5281/zenodo.5791191 ^40^ This project contains the following underlying data:

- data_clinical_patient.txt
- data_clinical_sample.txt
- data_CNA.txt
- data_CNA_subset_4000_genes.txt
- data_mutations_extended.txt

Data are available under the terms of the Creative Commons Attribution 4.0 International license (CC-BY 4.0). 

### Extended data

Zenodo: GNOSIS: an R Shiny app supporting cancer genomics survival analysis with cBioPortal, https://doi.org/10.5281/zenodo.5788544 ^41^

This project contains the following extended data:

- GNOSIS_Tab_1_Input_Files.mp4
- GNOSIS_Tab_1_Input_Files_with_Subtitles.mp4
- GNOSIS_Tab_2_Exploratory_Tables.mp4
- GNOSIS_Tab_2_Exploratory_Tables_with_Subtitles.mp4
- GNOSIS_Tab_3_Recode_Subset.mp4
- GNOSIS_Tab_3_Recode_Subset_with_Subtitles.mp4
- GNOSIS_Tab_4_Exploratory_Plots.mp4
- GNOSIS_Tab_4_Exploratory_Plots_with_Subtitles.mp4
- GNOSIS_Tab_5_KM_Plots.mp4
- GNOSIS_Tab_5_KM_Plots_with_Subtitles.mp4
- GNOSIS_Tab_6_Association_Tests.mp4
- GNOSIS_Tab_6_Association_Tests_with_Subtitles.mp4
- GNOSIS_Tab_7_Cox_Models.mp4
- GNOSIS_Tab_7_Cox_Models_with_Subtitles.mp4
- GNOSIS_Tab_8_Adjusted_Survival_Curves.mp4
- GNOSIS_Tab_8_Adjusted_Survival_Curves_with_Subtitles.mp4
- GNOSIS_Tab_9_Survival_Trees.mp4
- GNOSIS_Tab_9_Survival_Trees_with_Subtitles.mp4
- GNOSIS_Tab_10_Maftools.mp4
- GNOSIS_Tab_10_Maftools_with_Subtitles.mp4
- GNOSIS_Tab_11_Download_Code_Log.mp4
- GNOSIS_Tab_11_Download_Code_Log_with_Subtitles.mp4

Videos are available under the terms of the Creative Commons Attribution 4.0 International license (CC-BY 4.0).

## Software availability

Source code available from: https://github.com/ Lydia-King/GNOSIS

 Archived source code as at time of publication: https://doi.org/10.5281/zenodo.5788595

License: MIT
