# Supplementary material for: GNOSIS: an R Shiny app supporting cancer genomics survival analysis with cBioPortal
Source: HRB Open Res. 2022 Sep 12;5:8. Originally published 2022 Jan 18. [Version 2] doi: 10.12688/hrbopenres.13476.2 (PMC9051584; doi:10.12688/hrbopenres.13476.2)
Supplement: Peer review history

# Peer review: Reviewer response for version 1

**DOI:** 10.21956/hrbopenres.14690.r31939

**Published:** 2022-04-28

**Authors:** Tiago C. Silva

**Affiliations:** 1 Division of Biostatistics, Department of Public Health Sciences, Miller School of Medicine, University of Miami, Miami, FL, USA

King *et al*. developed GNOSIS (GeNomics explOrer using StatistIcal and Survival analysis in R), an open-source R Shiny app that provides an easy non-coding way to analyze clinical and cancer genomic data. With data in the same format as the ones provided by cBioPortal, the users can perform EDA (Exploratory Data Analysis), survival analysis, and mutation analysis.

 Overall, the graphical user interface is well designed and straightforward to use, and tutorial videos are available as documentation. Using the provided example dataset, I was able to reproduce most of its functionalities. Below are some comments and suggestions.

**Major comments:**

- Using the data_mutations_extended.txt file the Maftools summary tab is giving an error in the shinyapps.io version.
- If I upload the data and jump directly to exploratory plots, it asks to calculate CNA Scores. You should still be able to use this tab without calculating CNA Scores. Also, if you calculate as “single gene”, it seems this tab does not work.

**Minor comments:**

- In the Survival tree tabs and the corresponding survival curves panel, the plot could be improved by removing the gray background and the addition of the p-value.
- I was not able to understand the purpose of the “The exploratory tables” tab. Is it only selecting columns? If so, I believe this functionality is not useful and distracting, users could simply open it in spreadsheet software (MS. Excel, google sheets, open office) and have more freedom to filter.
- Providing a vectorized version of plots would be very useful since PNGs are not modifiable.
- Filtering the options in the selection of variables would make the GNOSIS easier to use.
- The Association test tab could be improved with the addition of the compareGroups package.

Are the conclusions about the tool and its performance adequately supported by the findings presented in the article?

Yes

Is the rationale for developing the new software tool clearly explained?

Yes

Is the description of the software tool technically sound?

Yes

Are sufficient details of the code, methods and analysis (if applicable) provided to allow replication of the software development and its use by others?

Yes

Is sufficient information provided to allow interpretation of the expected output datasets and any results generated using the tool?

Partly

Reviewer Expertise:

Software engineering, bioinformatics, data analysis, cancer

I confirm that I have read this submission and believe that I have an appropriate level of expertise to confirm that it is of an acceptable scientific standard.

# Response

**Published:** 2022-06-10

**Authors:** Aaron Golden

**Affiliations:** National University of Ireland, Galway, Ireland

**Reviewer Point P 2.1** – *Using the data mutations extended.txt file the Maftools summary tab is giving an error in the shinyapps.io version.*

**Reply:** We thank the reviewer for bringing this to our attention, the issue is now fixed

**Reviewer Point P 2.2** – If I upload the data and jump directly to exploratory plots, it asks to calculate CNA Scores. You should still be able to use this tab without calculating CNA Scores. Also, if you calculate as “single gene”, it seems this tab does not work.

**Reply:** We thank the reviewer for pointing this out. Users do have the ability to create exploratory plots for the clinical variables and CNA calls for each patient separately, once the respective files are uploaded on their own. However, when users upload the clinical and CNA data together, the two files are integrated and merged on the column PATIENT ID. In this case, rather than being able to select specific patients, the user is asked to select genes to interrogate. As a result, the number of options available for users to select increases significantly (for example from 2000 to ¿ 20000) and so to avoid overwhelming both the user and the App, users are asked to calculate CNA Scores for each patient or to choose a number of individual genes to interrogate. This means that instead of having upwards of 22,000 genes to choose from, users can choose to create a whole genome CNA Score or pick specific genes of interest to analyse. We hope the above explanation sufficiently explains why the user is asked to calculate CNA Scores/Select single genes before downstream analysis when both the clinical and CNA data are uploaded and addresses the reviewer’s concerns.

**Reviewer Point P 2.3** – *In the Survival tree tabs and the corresponding survival curves panel, the plot could be improved by removing the gray background and the addition of the p-value.*

**Reply:** We agree that the survival curve plots within the Survival Trees Tab, and indeed all plots throughout the App, could be made clearer by removing the gray background. As a result, the back ground colour for all plots has been changed to white. In terms of adding the p-value to the survival curves corresponding to survival tree node, this function is already available and can be implemented using the “Display P-value” button within the right box sidebar.

**Reviewer Point P 2.4** – *I was not able to understand the purpose of the “The exploratory tables” tab. Is it only selecting columns? If so, I believe this functionality is not useful and distracting, users could simply open it in spreadsheet software (MS. Excel, google sheets, open office) and have more freedom to filter.*

**Reply:** We thank the reviewer for this feedback. The purpose of the “The exploratory tables” tab is to enable further exploration of user selected columns. While it technically is only selecting columns to view in a clearer, less cluttered way, and the users could indeed use spreadsheet software for this purpose, we think this tab is useful. It allows users to run all steps in their analysis in one place and gives them the opportunity to access the R code used to select columns via a downloadable R script. As a result, we have decided to keep this tab in the App.

**Reviewer Point P 2.5** – *Providing a vectorized version of plots would be very useful since PNGs are not modifiable.*

**Reply:** We thank the reviewer for this extremely useful suggestion and in response changes have been made to the App. Users are now given the option to download the plots in both .png and .svg format.

**Reviewer Point P 2.6** – *Filtering the options in the selection of variables would make the GNOSIS easier to use.*

**Reply:** Filtering of variables in the select boxes is already implemented. Users can remove the “None Selected” input in the select box and type in a letter or word to filter variable names. This makes it easier to use and means users can search for specific variables or reduce the number of variable names shown in select input boxes.

**Reviewer Point P 2.7** – *The Association test tab could be improved with the addition of the compareGroups package*.

**Reply:** We thank the reviewer for this suggestion and have added a section where users can utilise the basic functions of the compareGroups package.

# Peer review: Reviewer response for version 1

**DOI:** 10.21956/hrbopenres.14690.r31288

**Published:** 2022-03-15

**Authors:** Jessica C. Mar

**Affiliations:** 1 Australian Institute for Bioengineering and Nanotechnology, The University of Queensland, Brisbane, Qld, Australia

King *et al*. have developed an open-source tool using R Shiny that facilitates analyses on largescale cancer genomic data and related clinical variables. While the analyses that this tool implements are straightforward and routine, the utility of this tool is that it is easy to use and removes the need for advanced programming skills. Overall, this is a thoughtfully designed tool that succeeds in its aim to make this kind of data analysis accessible to everyone.

 With regards to GNOSIS' role in generating robust and reliable statistical analyses, are there clear definitions or recommendations that users are directed to, to ensure that the statistics are being used appropriately with the input data? For example, on p7, the Chi-squared test is being used "with sufficient cell size" - is this defined somewhere in the tool? Similarly when working with survival analysis, it is necessary to ensure that the data meets regulatory assumptions, is there a note somewhere so that users can assess this?

 Another aspect which the authors could comment on is what are some of the other alternatives or similar tools to GNOSIS that are currently out there? Either to appreciate the utility of GNOSIS or to give users options if they are looking for something that may sit outside of GNOSIS' scope?

Are the conclusions about the tool and its performance adequately supported by the findings presented in the article?

Yes

Is the rationale for developing the new software tool clearly explained?

Yes

Is the description of the software tool technically sound?

Yes

Are sufficient details of the code, methods and analysis (if applicable) provided to allow replication of the software development and its use by others?

Yes

Is sufficient information provided to allow interpretation of the expected output datasets and any results generated using the tool?

Yes

Reviewer Expertise:

Bioinformatics, Computational Biology, Biostatistics

I confirm that I have read this submission and believe that I have an appropriate level of expertise to confirm that it is of an acceptable scientific standard.

# Response

**Published:** 2022-06-10

**Authors:** Aaron Golden

**Affiliations:** National University of Ireland, Galway, Ireland

**Reviewer Point P 1.1** – *With regards to GNOSIS’ role in generating robust and reliable statistical analyses, are there clear definitions or recommendations that users are directed to, to ensure that the statistics are being used appropriately with the input data? For example, on p7, the Chi-squared test is being used ”with sufficient cell size” - is this defined somewhere in the tool? Similarly when working with survival analysis, it is necessary to ensure that the data meets regulatory assumptions, is there a note somewhere so that users can assess this?*

**Reply:** We thank the reviewer for bringing this up and agree that it is extremely important that the statistical tests are being used and interpreted appropriately. To aid users in deciding what test is most appropriate, if the assumptions of the test are met, how to interpret the results and also how to run a basic analysis in R we have included an information button in certain tabs which include links to helpful websites. These changes can be seen throughout the App, mainly in the Kaplan-Meier Plots tab, the Association Tests tab, the Cox PH Models tab and the Survival Trees tab, and we hope this will make it easier for users to perform a robust and statistically sound analysis.

**Reviewer Point P 1.2** – *Another aspect which the authors could comment on is what are some of the other alternatives or similar tools to GNOSIS that are currently out there? Either to appreciate the utility of GNOSIS or to give users options if they are looking for something that may sit outside of GNOSIS’ scope?*

**Reply:** We thank the reviewer for suggesting this and in response have amended the manuscript to include other alternatives or similar tools to GNOSIS that users may find helpful.
